# The Reduced Multi-Dimensional Health Literacy Scale: A First Bilingual Validation Using RASCH Analysis

**DOI:** 10.3390/ijerph22111746

**Published:** 2025-11-19

**Authors:** Heike Wieser, Waltraud Tappeiner, Fabio Vittadello

**Affiliations:** 1 Claudiana Research, University Center for Health Professions Claudiana, 39100 Bolzano, Italy; 2 Explora—Research and Statistical Analysis, 35010 Padova, Italy

**Keywords:** reduced health literacy scale, validity, reliability, Rasch analysis, type 2 diabetes

## Abstract

This study aimed to evaluate the reliability and validity of the reduced health literacy scale (HLS) of Ishikawa, as used in translated German and Italian versions. Methods: The reduced HL scale was applied to a convenience sample of 100 persons with type 2 diabetes (DM2) in a province of Northern Italy. Rasch analysis was used to evaluate the psychometric measures of the nine items, regarding communicative and critical HL. Results: Person and item reliability was 0.87 for the German and 0.72 for the Italian version. Correlation values ranged from 0.55 to 0.76 (German version) and from 0.48 to 0.70 (Italian version). MNSQ values INFIT and OUTFIT expressed generally positive results. Possible redundancy was observed for one item in the German and Italian version, respectively, and in one for both versions. The values obtained in the analysis indicate a good fit, and no item had to be changed. In the German version, about 14% of persons demonstrate a high HL level (10% for the Italian version), while 39% expressed a low level of HL (56% for the Italian version). Conclusions: No particularly critical items of the reduced HL scale emerged in both versions applying Rasch analysis. The use of the reduced scale (only nine items) has the advantage of being integrated into longer questionnaires and into more structured assessment instruments. Furthermore, it could also be used as a rapid screening tool, allowing health professionals to address patients and their HL needs in a more targeted way. However, more psychometric testing with a larger sample is required.

## 1. Introduction

Health literacy is the degree of the capability of an individual to deal with health-related issues (understood as the extent of its ability to find, understand, evaluate and use health related information) [1]. More recently, it has also been seen as a responsibility of the organizations to provide an environment which is sensitive to the needs of patients/users, so that they can navigate the healthcare system in an adequate and easier manner [2]. The availability of short instruments for measuring different dimensions of clients’/patients’ health literacy in routine healthcare, but also the allocation of resources and time, is crucial to ensure that health professionals can assess and improve their clients’ HL. Recently, a survey on health professionals’ preparation in terms of professional health literacy (pHL) highlights that it is of particular importance to be able to assess and understand their patients’ HL levels [3].

Ishikawa et al. [4] were among the first to develop and apply a scale that measured the multidimensional construct of health literacy (HL), as proposed by Nutbeam [5], including dimensions such as the communicative (ability to interact) and the critical (ability to verify/check), along with the functional or basic (ability to read and write) HL. People with an adequate or good level of health literacy have greater abilities to search for health-related information, to understand it, and to adapt and apply it to their own situation compared to persons with lower HL [1,6].

There are other HL scales [7,8] or questionnaires [9,10] published measuring more dimensions of HL. Among them, the HLS of Ishikawa [4], according to Lee et al. [11], was the most appropriate instrument for persons with diabetes, due to its structure and content. The HLS suits measuring multidimensional HL. At the same time, to date there are no experiences of administering this scale in Italy. While several translations/re-validations of the HL scale exist, most applied classical test theory (CCT), with translated versions available, for example, in Dutch [12], German [13], Korean [7], English/American [14], Serbian [15], and Chinese [16].

In a previous study, the authors of the present article gained insights into the extent to which key healthcare professionals (HCP) in a bilingual (German and Italian) Northern Italian region address and target nutritional and physical activity behaviours in people with type 2 diabetes in routine care. In that study, 100 persons with type 2 diabetes were included and analysed regarding their readiness towards change in their nutritional and physical habits. Additionally, their critical and communicative HL was assessed by applying this part of the multidimensional HL scale of Ishikawa [17].

While the German version of the original 14 item HL scale [13] was available and validated with common test theory, the Italian version had to be developed anew.

However, for the first time, both scales (German and Italian) were used as a reduced version (nine items), so there was a need to validate their measurement properties.

Rasch analysis [18,19] is known as a statistical procedure for constructing and validating instruments. Based on ordinal data, such as questionnaire responses, it enables the determination of objective quantitative measures by simultaneously categorizing participants (by ability or suitability) and items (by difficulty) on a single scale. This approach ensures greater accuracy and validity in scoring because it verifies the unidimensionality of the scale, identifying dysfunctional items and allowing valid comparisons between respondents, even when different item sets are present. Due to these features, Rasch analysis is an excellent tool for validating data, even from small samples.

Therefore, this study aims to present the measurement properties of the reduced HL scale (nine items) of Ishikawa et al. [4], obtained with Rasch analysis, as applied to the above-mentioned convenience sample. In this way, we also aim to contribute to the availability of validated short scales that can be used in a busy clinical environment without overloading patients and healthcare professionals.

## 2. Materials and Methods

### 2.1. Sample and Data Collection

The data were obtained from a survey conducted in 2019 on a convenience sample of persons who had type 2 diabetes and members of a local patients’ association (n = 100). A total of 64 of them answered in Italian and 36 in the German language.

Participation was completely voluntary, and consent was given by returning the questionnaire.

### 2.2. The Instrument and Its Adaptation and Translation Process

To measure HL of the study participants, the HL scale developed by Ishikawa et al. [4] was chosen and applied due to its shortness, measuring three dimensions/factors (functional, communicative, and critical) of HL; further, the scale had already been evaluated in persons with type 2 diabetes. The original scale had undergone reliability testing and EFA (exploratory factor analysis). Its total scale reliability (Cronbach’s alpha) was α = 0.78, and all item total correlations were 0.60–0.85 (communicative HL) and 0.63–0.83 (critical HL), only reporting a significant positive association for communicative and critical HL (r = 0.52, *p* < 0.001) [4].

The complete HL scale (14 items) consists of five items evaluating the functional HL of instructions/leaflets in terms of readability and understandability to patients. Five items evaluating the capabilities and practices of communicative HL refer to the collection and comprehensibility of the information searched for. Lastly, the other four items assessed the critical HL, such as the applicability to the own situation, and the validity and credibility of the information found.

The research team decided to use a reduced version (nine items) of the multidimensional HL scale [4], omitting the functional HL items, as the interest was in whether and how patients searched for information to make health decisions, if they understood it, if they applied it to their daily life, and if they asked themselves if this information was reliable and valid. These aspects reflect the content of the communicative and critical components of the HLS.

As the aim was to gather knowledge about how people with chronic disease live with their situation, focusing on nutritional and physical activity behaviour, the communicative and critical HL was considered to be more relevant. Additionally, the authors tried not to overload the questionnaire used in that survey.

The full version of the HL scale used had already been translated and validated in the German language (α = 0.77) by Dwinger et al. [13], but was, at the time of the study, unavailable in the Italian language.

The scale was translated and adapted according to the ISPOR principles as outlined by Wild et al. [20], applying criteria such as receiving permission to use the instrument from the developers and using native speakers (Italian) for the forward translation, as well as producing two independent versions. As Ishikawa had suggested to us that Dwinger et al. [13] had translated the instrument into the German language, we used this validated version as a model (i.e., it served as the scale upon which we relied and upon which we based our translation into Italian). The two translations produced by two independent Italian-speaking healthcare professionals (nurse, dietician) in the following step were compared and reviewed by another Italian-speaking healthcare professional (physician). Disagreements and differences were resolved by reaching a consensus with the principal investigator. Afterwards, the Italian version was translated back into German.

Both versions (German and Italian) were used for cognitive debriefing afterwards, involving eight persons from different healthcare professions, such as physicians, nurses, dieticians, and psychologists. Every single item was rated for relevance on a 4-point Likert scale (from “not at all relevant” to “highly relevant”), and if the items were clear and understandable (yes/no).

### 2.3. Applying Rasch Analysis

Rasch analysis offers a good possibility to evaluate the measurement properties, even in small samples, and offers advantages over classical test theory, such as simultaneously examining the items and respondents’ performance as it quantifies generally latent psychological factors. This method/approach transforms ordinal data into linear measures with equal-interval units called logits, which are used to describe the measures of both individuals and items [21]. This transformation of raw scores into a linear continuum allows us to measure the ability of a person and the difficulty of the items.

In order to test the reduced HLS with its communicative and critical HL dimensions, the following research questions were asked: “How does the reduced Ishikawa HL scale perform in the German- and Italian-speaking sample described above?” More detailed research questions were the following:

- To what extent do the single items of the reduced HL scale contribute to measuring the whole construct? (Model fit/misfit).
- How are item and person difficulty distributed? (Wright map or person/item map).
- Are there items that reduce the validity of the scale? (Item polarity).
- How well can the reduced HL scale distinguish between people with lower and higher HL? (Person and item separation index).

Table 1 presents the indices applied in Rasch analysis and the corresponding reference values.

Concerning the items’ dimensionality, the INFIT and OUTFIT MNSQ values give information about how suitable an item is to measure the validity [18] and values that are outside of the acceptable range are not fulfilling the expected fit to the model. Model underfit (values < 0.5) reduces the validity of the model and requires further investigation to determine the reason for the underfit. On the contrary, model overfit (values > 1.5) could result in a misinterpretation that the model worked better than expected, but it does not reduce the validity of the model [18]. Outfit mean square values (MNSQ), outfit z-standardized values (ZSTD), and point measure correlation (PTMEA-CORR) are three criteria to be used for assessing the item fit, as suggested by Boone [21] and Bond and Fox [18]. However, an item is misfitting if all the three criteria are out of the fit range. Particular attention was paid, following Linacre [26], to the values of the INFIT MNSQ parameter, which detects possible inconsistencies between the most important items in measuring the construct.

The study obtained ethical approval from the South Tyrolean Health Trust’s Ethics Committee (no. 08/2018, 17 January 2018). For descriptive analysis, we used SPSS Statistics version 29.0.1.0., while for Rasch analysis, the software Winsteps version 1.0.0. [27] was applied.

## 3. Results

This section presents the characteristics of the sample of respondents, the descriptive results of the items that make up the reduced HL scale, and the values of the indicators calculated through Rasch analysis. All reported results are distinguished according to the language of the scale: German (n = 36) and Italian (n = 64).

### 3.1. Sample Characteristics

Table 2 presents the main characteristics of respondents according to their language: German or Italian. The sample consisted of 57 men and 40 women, and gender was missing in three cases, as it was the weight. The great majority of the respondents (78%) indicated to be diagnosed more than 10 years ago, while nearly half reported to have attended a diabetes education/training session (49%). One out of three had diabetes complications such as neuropathy, nephropathy, retinopathy, and diabetic foot, and two in three (66%) were overweight or obese. We noticed significant differences between the German and Italian participants for those having complications: they were differing in BMI classes and living in an urban area, as reported in Table 2.

### 3.2. HL Scale: Items and Attitudes

**Figure 1:**
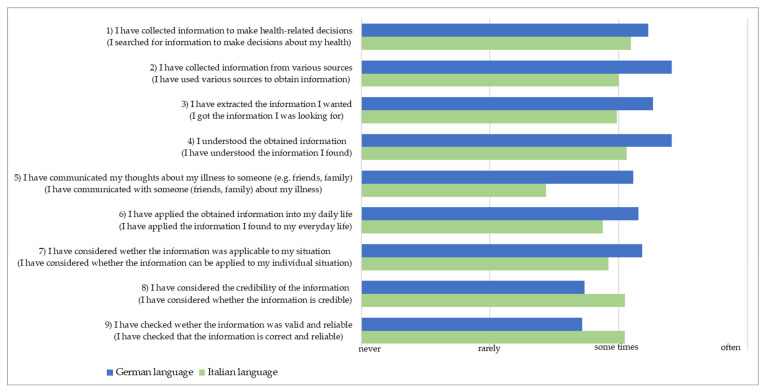
Items of the reduced HL scale: mean scores according to the language of participants.

Figure 1 shows the average values assigned to the single items (ordered from item 1 to item 9) by the German and Italian participants. The item descriptions are those of the original version of the instrument, while the contents of the administered items obtained through the back-translation procedure from Italian and German are given in brackets.

As can be seen in Figure 1, respondents of the German version (blue bars) recognised their behaviour in the actions expressed by the items with a fairly high frequency, except for items 8 and 9, about reliability and correctness of the retrieved information. Respondents of the Italian version (green bars) show a generally lower frequency of application, particularly regarding their experience of discussing their health condition with other people (item 5). Differently, the assessment of the reliability and correctness of the information found for item 8 and 9 is higher than in the sample who filled out the German version.

### 3.3. Principal Component Analysis

The principal component analysis (PCA) of the residuals was conducted to examine uni-dimensionality. The observed variance explained in the Italian version of the scale was 37.1%, similar to the expected variance of 37.8% in the model. The unexplained variance in the first contrast was 18.1% (Eigenvalue: 2.6) and less than the variance explained by the items (28.8%). Instead, for the German version, the measure was 65.6%: close to the expected variance in the model (67.7%). The unexplained variance in the first contrast was 11.5% (Eigenvalue: 3.0) and less than the variance explained by the items (33.3%). Considering that the scale consists of only nine items, the values reported above are acceptable to prove uni-dimensionality, according to Bond and Fox [18] and Linacre [27].

### 3.4. Reliability Measures and Separation Index

For the reduced HL scale, the obtained person and item reliability was equal to 0.87 for the German and 0.72 for the Italian version of the scale. According to George and Mallery [28], these values demonstrate an acceptable reliability for both versions of the scale. The separation index provides the extent to which a scale differentiates between different respondents, according to the value of the construct analysed. The item separation index obtained for the German version was 1.69, indicating the need for a bigger sample according to Rasch guidelines. The person separation index obtained for the German version (2.57) does outline that the item number is enough [18], while for the Italian version, both the item separation index (1.49) and the person separation index (1.60) were below the cutoff values presented in Table 1.

### 3.5. Item Polarity

Item polarity (expressed by the point measure correlation, PTMea) is an indicator of how well a single item measures the construct to be measured. According to Bond and Fox [18], all values must be positive (above 0); a value of up to 0.35 indicates a low correlation, a value from 0.36 to 0.67 indicates a moderate correlation, and a value between 0.68 and 1 indicates a high correlation. In our study, we obtained values ranging from a moderate to a high correlation, as shown in Table 3 for the German (0.55–0.76), and only moderate values for the Italian version (0.48–0.70).

Table 3 shows, for the German version of the scale, a high correlation for items 4 (0.76), 8 (0.73), 2 (0.70) and 3 (0.69), while all other items had a moderate correlation, whereas for the Italian version, only item 6 showed a high correlation (0.70), while all other items had a moderate correlation.

### 3.6. Item Fit/Misfit Measures

Item fit and misfit measures are reported as INFIT and OUTFIT mean square value (MNSQ), as indicated in Table 1, and should be close to 1.0 with an acceptable range of 0.6–1.4, or 0.7–1.3 for a single item, according to Linacre [26]. Furthermore, according to Smith et al., these MNSQ values seem to not be sample-size-sensitive for polytomous data, as was the case in this study (Likert scale) [29].

In Table 4 and Table 5, we report the results about the fit/misfit measures obtained for both versions of the reduced HL scale.

INFIT and OUTFIT values reported as a mean square value (MNSQ) should be close to 1.0, with a desired range of 0.7–1.3 for a single item in small samples [26].

The INFIT and OUTFIT MNSQ values that are outside of this interval are interpreted as less than the expected fit to the model. Model underfit (values < 0.5) reduces the validity of the model and requires further investigation to determine the reason for the underfit. Regarding item fit, the MNSQ values INFIT and OUTFIT were assessed in the study. Values smaller than 0.7 are interpreted as a pattern of responses, which are too predictable [18], suggesting that the items’ content might be redundant with that of other items. This was observed for item 4 (“I understood the obtained information”) of the German version and for the Italian version, as well as for item 6 (“I have applied the obtained information into my daily life “) of the Italian version. An overfit (values > 1.3) was observed only for item 5 (“I have communicated my thoughts about my illness to someone (e.g., friends, family)”) for both languages.

### 3.7. The Wright Map

**Figure 2:**
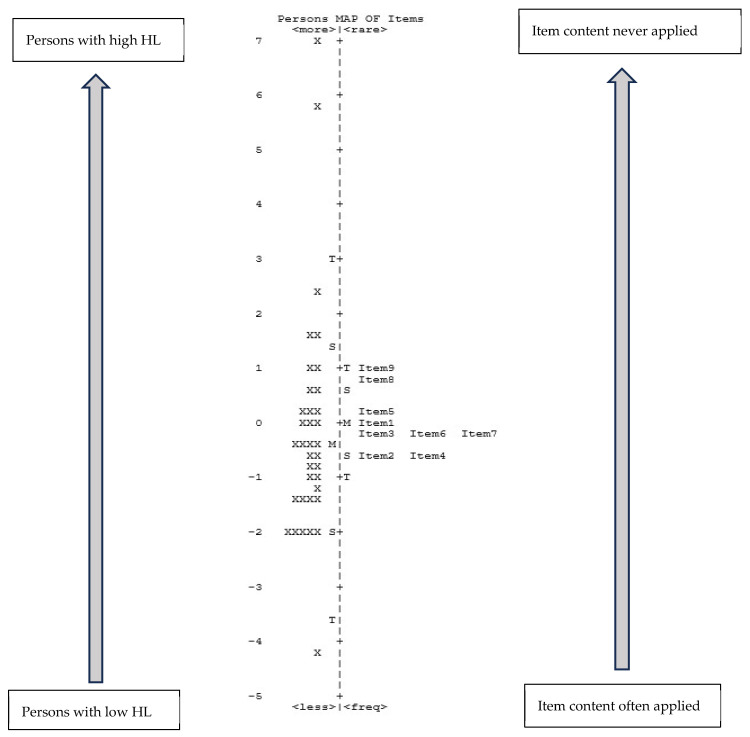
German version of the reduced HL scale (n = 36). Legend: The letters on the left and right side of the ruler (dotted line) indicate the mean value (=M), one standard deviation (=S), and two standard deviations (=T); X = one participant.

**Figure 3:**
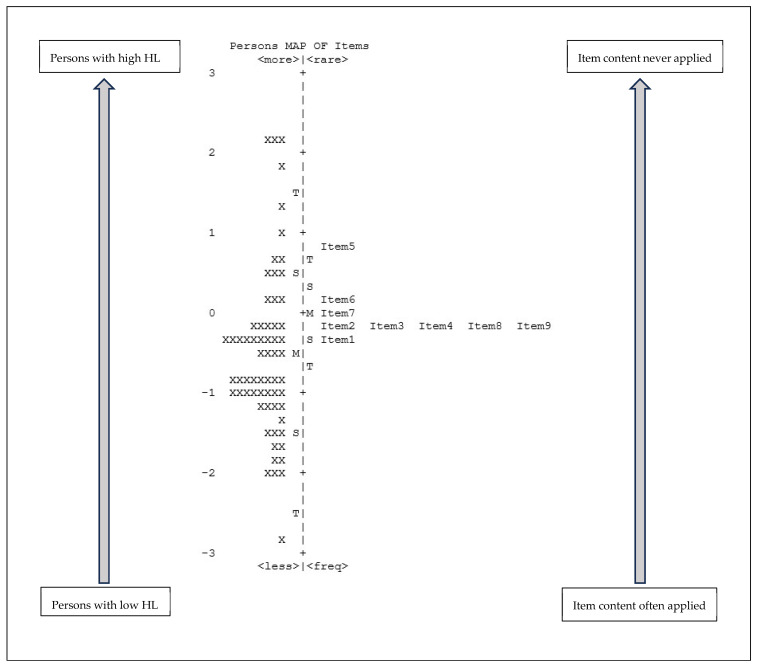
Italian version of the reduced HL scale (n = 64). Legend: The letters on the left and right side of the ruler (dotted line) indicate the mean value (=M), one standard deviation (=S), and two standard deviations (=T); X = one participant.

The Wright map (person/item map) describes how respondents answered the single items, i.e., how able they were to gather, assess, and apply relevant health information (HL), expressed as a person’s ability. While item difficulty is expressed/described by the level of agreement, items with a higher measure (more frequently performed) are items that were harder to agree with. The difficulty of the individual respondent is shown on the left-hand side of the dashed line (see Figure 2 and Figure 3), and the difficulty of the item is shown on the right-hand side. Persons with low HL are displayed on the bottom of the scale, while the more difficult items to answer are displayed at the top of the scale.

As shown in Figure 2, about 14% of persons demonstrate to have a high HL level (highest rated item), while 39% expressed a low level of HL (lowest rated item). The items that were most difficult to apply were item 9 and item 8, while the easiest items were item 2 and item 4.

The items that appear on the same line possibly indicate redundancy, as they look very similar to each other. As Figure 2 demonstrates, this happens for the German version in several situations: item 2 with item 4 and item 3 with items 6 and 7.

The Wright map obtained for the Italian version (Figure 3) shows that about 10% of persons had a high HL level, while for 56% it was low. The item with the lowest rating was item 5 (rarely applied), in contrast to item 1 (often applied). However, as in Figure 2, some items appear on the same line, indicating a possible redundancy. As Figure 3 illustrates, this has occurred for items 2, 3, 4, 8, and 9.

For both groups, the mean value of rating the items (at the left of the dotted line) and the mean value of HL-level (at the right of the dotted line) are close to each other. The list of items proposed with the scale is appropriate for the sample surveyed, being neither too simple nor too complicated [30].

## 4. Discussion

This study aimed to present results about the measurement properties of the reduced HL scale (nine items) of Ishikawa et al. [4], obtained through Rasch-analysis.

The mean scores of the items (Figure 1) show a higher value for the vast majority (seven out of nine items) among German-speaking respondents than among Italian-speaking respondents. In this analysis, we refer to a higher HL value related to a better attitude towards the disease. Thus, lower subscale mean values are interpreted as assigning a lower importance to the disease, particularly in relation to communication with family and friends and the activity of searching for and evaluating the information. Interestingly, the Italian-speaking participants seem to be more attentive about appraising the credibility of the information they retrieved. This could be explained through cultural differences between the Italian- and German-speaking groups, which is routed in historical circumstances. For example, about 70% of all Italian-speaking citizens of the region live in the urban area.

Regarding the German version, the results indicate the need for a bigger sample, while confirming the number of items administered. In contrast, the separation indices for the Italian version express the need for both a larger sample and a larger number of items (person and item reliability).

Referring to the item validity, evaluated by PTMea values in Table 3, values ranging from a moderate to a high correlation were obtained for the German version (0.55–0.76) and only moderate values were obtained for the Italian version (0.48–0.70). Based on these results, it appears that the set of items measures the construct better in the German version than in the Italian version.

Regarding item fit, MNSQ values INFIT and OUTFIT, generally positive results were obtained. Possible redundancy was observed for item 4 (“I understood the obtained information”) of the German version and for the Italian version, as well as for item 6 (“I have applied the obtained information into my daily life “) of the Italian version. For both languages, only item 5 (“I have communicated my thoughts about my illness to someone (e.g., friends, family)” expressed an overfit value. This could be an indication that the content of this item is too unexpected/unpredictable. The values obtained in our analysis indicate a good fit, and no item had to be changed.

As the instrument had to be administered in both languages (German and Italian), in line with the bilingual nature of the sample, we decided to use the validated version in German that was already available in the literature [13]. This version of the scale, which Dwinger et al. provided to us, was then translated into the Italian language to ensure maximum consistency (item equivalence) with the items expressed in German. But, looking more closely, some discrepancies with the original English version [4] were identified. In general, the original items are formulated in a less engaging/activating way for the respondents compared to the German version. The greatest linguistic difference was observed for item 5 (“I have communicated with someone (friends, family) about my illness”). Even items 4 and 6 in both languages do not correctly reflect the contents of the original items. Based on the German version [13], these two items stated, respectively, “I have understood the information I found” (item 4) and “I was able to transfer the information I found to my everyday life” (item 6), unlike in the original version, which stated, “I have understood the obtained information” (item 4) and “I have applied the obtained information into my daily life” (item 6). The wording of item 4 in the translated versions (German, Italian) addresses maybe a more active role of the study participant/patient than in the original version (expressed by “information I found” versus “the obtained information”).

This shift towards a more active information retrieval attitude/practice of people could be partially explained because of the great improvement and ease that has taken place in accessing information (through the world wide web and the availability of smart phones) since the scale was developed and introduced [4]. These subtle differences between the two versions, especially for item 6, may have influenced the interpretation of the item, and consequently, its frequency of application. The over and underfit of some items in the Italian version could therefore be explained by their formulation in the reduced HL scale, as a tendency towards redundancy is observed for some items, especially in the Italian version. However, no item performed so badly as to suggest its exclusion from the reduced HL scale.

Looking at the Wright map, the distribution of the items according to the variability of their rating shows an appreciable concentration, narrower than the distribution of the respondents. This imbalance could be interpreted as a limited ability to measure HL with this scale. This representation supports the potential redundancy of some items. We noticed a concentration of the items around the mean value along the main axis of the Wright map, which may suggest a certain uniformity in the perception and interpretation of the items. Furthermore, as discussed above, there seems to be an overlap of some items (to which we argue there is not) and they could therefore be perceived as having the same meaning. This similarity was found for items 2, “I have collected information from various sources”), 3 (“I have extracted the information I wanted”), 4 (“I understood the obtained information”), 8 (“I have considered the credibility of the information”) and 9 (“I have checked whether the information was valid and reliable”). For example, considering items 2 and 3, the overlap lies in the fact that both concern the activity of searching for information, but for the first item, the focus is the multiplicity of sources, while for the second item, it was the importance of the information. Therefore, although the Wright map suggests a possible duplication of some items, a careful evaluation of the items’ content highlights different meanings.

Considering the period when the HL scale was developed (2008), the source of information was almost exclusively represented by a health professional or perhaps a specialised journal. On the contrary, at the time of the study (2019), access to sources of information was much easier, due to the internet and social media, which allow for multiple information channels and sources. A challenge of today’s time is the evaluation of health information for patients/citizens, as pointed out, for example, by Diviani [31]. This challenge also exists for health professionals who need more specific preparation for dealing with communicative and critical HL aspects [3,32,33].

Other HL instruments/scales have been developed, such as the ‘All Aspects of Health Literacy Scale’ (AAHLS) by Chinn et al. [8], the ‘Comprehensive Diabetes Health Literacy Scale’ by Lee et al. [34], or the ‘Validation of the Assessments of Adult Health Literacy’ (AAHL-Adult) by Fleary et al. [35]. All authors wanted to integrate missing parts of previous measurement instruments, such as numeracy [34], or remediate a lack of test-based assessment instruments for measuring interactive and critical HL [35]. Unlike other studies, our aim was to test the validity of the reduced scale.

Limitations of the study: The great majority of participants in the study had a long history of their disease (more than 10 years). This could indicate that they have learnt to effectively manage their disease and to find ways to deal with related health information. It is therefore recommended to administer the scale to subjects with newly diagnosed type 2 diabetes. The fact that the sample were recruited as members of an association of diabetic patients could also be a bias in terms of increased awareness and attention towards the disease: thus, participants already had good health literacy. The differences in the sample characteristics are noteworthy and reflect the composition of the members registered in the patients’ organization, which may have contributed to a selection bias. The Italian questionnaire has lower validity results compared to the German version. These results should be further investigated to better understand the underlying factors (for example, cultural differences and sample characteristics).

Another limiting aspect of the study is the small sample size and the convenience sample, which both limit the generalisation of the results. It is therefore necessary to apply the reduced scale to larger samples to further validate its psychometric properties using both classical test theory and item response theory.

A strength of the reduced scale is its potential to be used in clinical practice (due to its shortness) for screening/classifying persons with different levels of HL, at least low versus high, as the Rasch analysis revealed, thus allowing health professionals to address patients and their needs in a more targeted way.

## 5. Conclusions

In conclusion, no particularly critical items of the reduced HL scale emerged in both versions when applying Rasch analysis. The use of the scale (only nine items) has the advantage of being integrated into longer questionnaires or being utilized in clinical practice, for example, for screening persons regarding their HL levels. Furthermore, it could also be used as a rapid screening tool, allowing health professionals to address patients and their HL needs in a more targeted way. However, more psychometric testing with a larger sample is required.

## Figures and Tables

**Table 1:** Overview of Rasch analysis indexes and reference values.

Criteria	Index	Reference Values
Person and item reliability	Person and item separation index (SE)	Separation index (SE) ≥ 2.0 and reliability value ≥ 0.80 (0.67–0.80 sufficient) [21].
Item validity	Item polarity	Point measure correlation (PTMea Corr.) > 0 (0.40–0.85) [21].
Item fit	Item dimensionality	Total mean-square INFIT and OUTFIT value within 0.7 to 1.30 [22,23].
Logits	The logit value indicates the logarithmic ratio between the probability of success and failure	A positive logit value means that the person’s ability is higher than the item’s difficulty → high probability of correct answer. A negative logit value means that the item is more difficult than the person’s ability → low probability of a correct answer [18,24].
Wright map	Graph displaying the distributions of respondents’ abilities and item difficulties on the same logit scale: on the right side, the items’ distribution is illustrated, and on the left side the person distribution is illustrated. A good scale should have a balanced coverage between the abilities of the respondents and the difficulties of the items [25].	

**Table 2:** Sample characteristics. All values are presented in numbers (n) and percentages (%). The *p*-values (Chi^2^ Test) refer to the evaluation of homogeneity of the sample.

Characteristics	German (n = 36)		Italian (n = 64)		*p* Value
	n	%	n	%	
diagnosed in the last 10 years	5	13.9	17	26.6	0.142
men	19	55.9	38	60.3	0.672
age under 70 years	14	40	19	30.2	0.323
attended diabetes education/training session	21	58.3	28	43.8	0.161
has diabetes complications	5	14.7	27	43.5	0.004
lives in an urban area	5	13.9	45	70.3	<0.001
normal weight (BMI)	14	38.9	17	27.9	0.028
overweight (BMI)	18	50	22	36.1	
obese (BMI)	4	11.1	22	36.1	

The dichotomized variables presented in Table 2 are as follows: men vs. women; urban versus rural area; diagnosis within last 10 years vs. more than 10 years; age < 70 vs. 70 and >70 years; having complications vs. no complications; participating in any diabetes training/education vs. not participating; and BMI class (normal weight = 18.5–24.9, overweight = 25–29.9, obese > 30).

**Table 3:** Item polarity for German and Italian versions of the reduced HL Scale.

Item Number	Item Description	PTMea Corr. Item	
		DE	IT
Item 1	I have collected information to make health-related decisions	0.64	0.57
Item 2	I have collected information from various sources	0.70	0.49
Item 3	I have extracted the information I wanted	0.69	0.50
Item 4	I understood the obtained information	0.76	0.58
Item 5	I have communicated my thoughts about my illness to someone (e.g., friends, family)	0.55	0.62
Item 6	I have applied the obtained information into my daily life	0.66	0.70
Item 7	I have considered whether the information was applicable to my situation	0.64	0.60
Item 8	I have considered the credibility of the information	0.73	0.50
Item 9	I have checked whether the information was valid and reliable	0.65	0.48

**Table 4:** INFIT and OUTFIT mean square values for the German version of the reduced HL scale (n = 36).

Item Number	Item Logit Measure	Model S.E.M.	INFIT		OUTFIT		Exact Obs.%	Match Exp%
			MNSQ	ZSTD	MNSQ	ZSTD		
Item 9	0.95	0.23	1.16	0.8	1.10	0.5	37.1	48.8
Item 8	0.79	0.23	0.94	−0.2	0.88	−0.4	54.3	50.0
Item 5	0.19	0.24	**1.45**	1.7	1.53	1.8	48.6	55.4
Item 1	0-.06	0.25	0.80	−0.7	0.96	0.0	51.4	56.0
Item 6	−0.13	0.26	1.10	0.4	0.92	−0.2	68.6	57.3
Item 7	−0.20	0.26	1.24	1.0	0.99	0.1	60.0	57.4
Item 3	−0.26	0.26	0.85	−0.5	0.80	−0.6	71.4	59.7
Item 2	−0.64	0.28	0.79	−0.7	0.68	−1.1	71.1	64.1
Item 4	−0.64	0.28	**0.49**	−2.1	0.46	−2.2	80.0	64.1
MEAN	0.00	0.26	0.98	0.0	0.93	−0.3	61.0	57.0
S.D	0.53	0.02	0.27	1.1	0.28	1.0	13.5	5.0

Note/legend: Model S.E.M. = model standard error of measurement; INFIT and OUTFIT MNSQ = information-weighted fit and outlier-sensitive fit mean square fit statistics; INFIT and OUTFIT ZSTQ = Z-standardized fit statistics; Exact Obs.% = exact observed percentage of responses compared with that predicted by the Rasch model; Match Exp% = match expected percentage of responses that match the model’s expectations, even if not exactly. The numbers in bold show the values out of range.

**Table 5:** INFIT and OUTFIT mean square values for the Italian version of the reduced HL scale (n = 64).

Entry Number	Item Logit Measure	Model S.E.M.	INFIT		OUTFIT		Exact Obs.%	Match Exp%
			MNSQ	ZSTD	MNSQ	ZSTD		
Item5	0.85	0.17	**1.34**	2.0	1.32	1.8	45.3	48.1
Item6	0.19	0.17	**0.59**	−2.7	0.58	−2.8	64.1	52.0
Item7	0.04	0.18	0.92	−0.4	0.94	−0.3	53.1	52.7
Item3	−0.09	0.18	1.08	0.5	1.00	0.1	64.1	53.0
Item8	−0.15	0.18	1.03	0.2	1.00	0.1	54.7	53.5
Item9	−0.15	0.18	0.98	−0.1	0.96	−0.1	57.8	53.5
Item2	−0.18	0.18	1.25	1.4	1.22	1.2	48.4	53.5
Item4	−0.18	0.18	**0.64**	−2.3	0.63	−2.3	65.6	53.5
Item1	−0.31	0.18	1.06	0.4	1.03	0.2	53.1	53.6
MEAN	0.00	0.18	0.99	−0.1	0.97	−0.2	56.3	52.6
S.D	0.33	0.00	0.23	1.5	0.23	1.4	6.8	1.6

Note/legend: model S.E.M. = model standard error of measurement; INFIT and OUTFIT MNSQ = information-weighted fit and outlier-sensitive fit mean square fit statistics; INFIT and OUTFIT ZSTQ = z-standardized fit statistics; Exact Obs.% = exact observed percentage of responses compared with that predicted by the Rasch model; Match Exp% = match expected percentage of responses that match the model’s expectations, even if not exactly. The numbers in bold show the values out of range.

## Data Availability

The raw data presented in this study are available on request from the corresponding author due to ethical restrictions.
